# Heating and storage of structured acylglycerols with succinyl-linked stigmasterol residue does not cause negative chemical or biological changes

**DOI:** 10.1038/s41598-023-48444-3

**Published:** 2023-12-04

**Authors:** M. Rudzińska, A. Grygier, A. Olejnik, K. Kowalska, D. Kmiecik, A. Chojnacka, W. Gładkowski, A. Grudniewska, R. Przybylski

**Affiliations:** 1 Faculty of Food Science and Nutrition, Poznań University of Life Sciences, Wojska Polskiego 28, 60-637 Poznań, Poland; 2 Department of Food Chemistry and Biocatalysis, Wrocław University of Environmental and Life Sciences, Norwida 25, 50-375 Wrocław, Poland; 3 Department of Chemistry and Biochemistry, University of Lethbridge, 4401 University Drive W, Lethbridge, AB T1K 3M4 Canada

**Keywords:** Drug discovery, Health care, Risk factors, Chemistry

## Abstract

Four structured acylglycerols with stigmasterol bonded by a succinyl linker were investigated and their stability were analyzed. Samples were heated to 60 °C and kept at that temperature to simulate storage, and to 180 °C to simulate frying conditions. The degradation of the synthesized compounds and formed derivatives was determined, and their cytotoxicity and genotoxicity on normal human cells from the digestive system was determined. Holding at 180 °C resulted in greater degradation of the compounds than holding at 60 °C. The most stable compound in each sample proved to be one with oleic acid in its structure—1,3-dioleoyl-2-stigmasterylsuccinoyl-sn-glycerol (DO2SSt) at 60 °C and 1,2-dioleoyl-3-stigmasterylsuccinoyl-sn-glycerol (DO3SSt) at 180 °C. These results indicate that the type of fatty acid in the molecule is more important than its position in the glycerol structure. None of the diacylmonostigmasterylsuccinoyl-sn-glycerols (DASStGs) before or after heating exhibited cytotoxic or genotoxic potential to small intestine and colon mucosa cells.

## Introduction

Plant sterols, also called phytosterols (PSs) are natural steroids widely found in different parts of plants, where they play an important role in cell membranes. They are found in different forms in plants, including free compounds, esters with fatty acids, steryl glycosides, and acylated glycosides^1^. The chemical structure of phytosterols is similar to cholesterol and they can cause total and low density lipoprotein cholesterol (LDL-C) to decrease in human plasma^2^. Additionally, they have been described as being able to increase insulin resistance and lipid metabolism as well as to reduce the risk of cancer, Alzheimer’s disease, and cardiovascular disease^1,3–5^. Their main sources in the human diet are vegetable oils, nuts, and cereals, and their consumption level is typically about100–500 mg/day^6–8^. However, the amount of plant sterols in natural food is too low to meet the supplementary recommendation of 2–3 g/day for adults^9^. Phytosterols are now being added with increasing frequency to food products as functional compounds. Since they are used as fatty acids esters, their daily intake in Europe is much lower than the recommended 2 g/day^10^. On the other hand, dietary exposure varies from 3.4 to 10.9 mg/(kg × day) in the case of baked food to 13.2 mg/day in potatoes fried in PS-enriched margarine^10^.

The US Department of Agriculture 2015–2020 report recommends cholesterol intake levels of below 300 mg/day, but data from Finland and the USA has shown that the amount ingested could be as high as 530–750 mg/day^11^. The bioavailability of plant sterols is very low and they are less than 5% absorbed in the digestive system, while cholesterol can be absorbed at 50–60%^12^. The intake of plant sterols therefore needs to be much higher than that of cholesterol, but such consumption increase the amount of their derivatives in the diet. Cholesterol and phytosterols are not stable during food processing or storage, instead forming a broad group of derivatives with negative effects on food quality and on the human body, including causing increases in the levels of oxidation products, volatile compounds, polar compounds, oligomers, and fragmented molecules^13^. Sterol oxidation can occur due to autoxidation, photosensitization, and enzymatic oxidation and is affected by the degree of unsaturation, temperature, light, water activity, and the presence of prooxidants, antioxidants, metals, and photosensitizers^10^. The bioaccessibility of phytosterol oxidation products (POPs) is much higher than that of free sterols and in the case of triol, α-epoxysitosterol, and 7β-hydroxysitosterol is as high as 60%^14^. The diversity of the biological effects of POPs include pro-inflammatory effects, cytotoxic effects, and the ability to affect cholesterol metabolism^15,16^.

The stability and safety of triacylglycerols (TAGs) depend on the length of the carbon chain of the esterified fatty acids, their unsaturation level, and their position in the glycerol backbone. TAGs with unsaturated fatty acids linked at the *sn*-2 position are more stable against oxidation than those linked at the *sn*-1 or *sn*-3 positions^17^. The study of Camacho Paez et al.^18^ showed that the introduction unsaturated fatty acids to the *sn*-2 position of glycerol by transesterification improves the absorption rate of essential fatty acids.

Structured acylglycerols are a class of glycerides with a specific molecular structure or function that chemically or enzymatically alters the composition or positional distribution of the glycerol skeleton^19^. In the present study, the oxidative stability and biological properties of four modified acylglycerols containing one molecule of stigmasterol were determined. The stigmasterol molecule was attached to the glycerol skeleton at the *sn*-2 or *sn*-3 position by a succinyl linker^20^. The distigmasterol-modified acylglycerols bonded with the succinyl linker and the products formed during their thermo-oxidation showed no cytotoxic or genotoxic activity to normal human cells and were more stable than compounds with stigmasterol bonded with glycerol backbone by carbonate linker^21^. There is a need to develop new compounds consisting of a phytosterol and acylglycerol molecule, which will show high stability during the production and storage of food products and will show activity in lowering cholesterol levels in human blood at the same level as free and esterified phytosterols. For the first time, new compounds that are hybrids of one molecule of phytosterol and diacylglycerol have been synthesized. Since the protective function of unsaturated fatty acids against phytosterol degradation has been previously described^22^, the synthesized new structural lipids will be characterized by significant stability during thermo-oxidation.

The aim of this work was to determine the thermal and oxidative stability of acylglycerols containing a phytosterol molecule in the *sn*-2 or *sn*-3 position and to investigate the effects of temperature and the position of stigmasterol in the glycerol skeleton on their degradation and toxicity.

## Results and discussion

**Figure 1:**
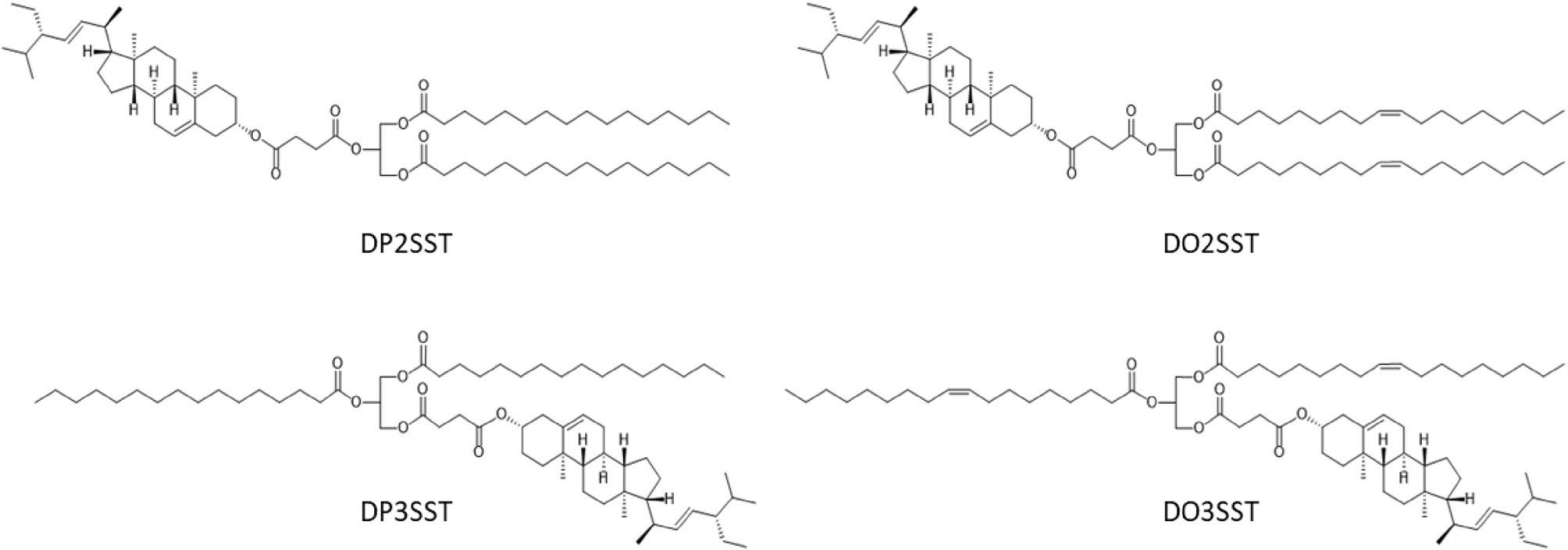
Chemical structures of diacylstigmasterylsuccinoyl glycerols (DASStGs): DP2SSt: 1,3-dipalmitoyl-2-stigmasterylsuccinoyl-*sn*-glycerol; DO2SSt: 1,3-dioleoyl-2-stigmasterylsuccinoyl-*sn*-glycerol; DP3SSt: 1,2-dipalmitoyl-3-stigmasterylsuccinoyl-*sn*-glycerol; DO3SSt: 1,2-dioleoyl-3-stigmasterylsuccinoyl-*sn*-glycerol.

Structured acylglycerols with two fatty acid residues (palmitic and oleic) located at the external positions of glycerol, and a stigmasterol residue linked to the internal position (in DP2SSt and DO2SST), as well as their asymmetric counterparts containing stigmasterol residue linked to the *sn*-3 position and fatty acid residues at *sn*-1 and *sn*-3 positions (in DP3SSt and DO3SST), were synthesized by Gładkowski et al.^20^.The stigmasterol was attached to the glycerol skeleton using a succinyl linker (Fig. 1). The stability and safety of these compounds after heating at temperatures simulating storage (the Schaal test) and frying was determined.

### Changes in DASStGs when holding at 60 °C

#### Stability of DASStGs

**Figure 2:**
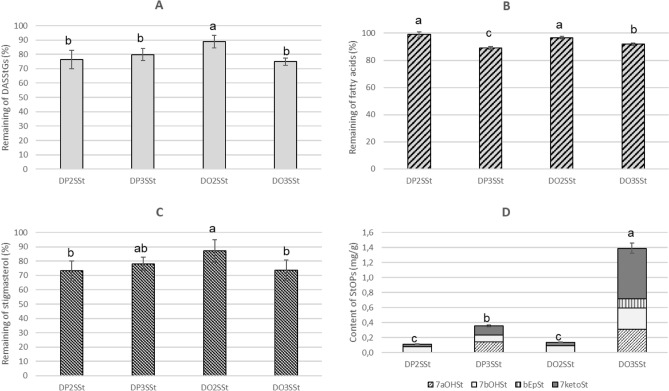
Amount of DASStGs remaining (**A**), fatty acid parts (**B**), stigmasterol parts (**C**) and the stigmasterol oxidation products formed after keeping DASStGs at 60 °C for 8 h: 7aOHSt: 7α-hydroxystigmasterol; 7bOHSt: 7β-hydroxystigmasterol; bEpSt: β-epoxystigmasterol; 7ketoSt: 7-ketostigmasterol; means with different letters differ significantly (*p* < 0.05).

The level of degradation in DASStGs kept at 60 °C for eight hours is presented in Fig. 2A. The degradation ranged from 11% in the case of DO2SSt to 25% in the case of DO3SSt. After heating, the amounts of DP2SSt and DP3SSt decreased by 20% and 24%, respectively. The results show that a new structured acylglycerol with stigmasterol at the *sn*-2 position and two oleic acid residues at the *sn*-1 and *sn*-3 positions was the most stable.

When the distigmasterol-modified acylglycerols were kept at 60 °C for 8 h, the lowest level of degradation was observed for compounds with oleic acid; this amounted to 5%, and when oleic acid was replaced by palmitic acid, the degradation increased to 8%^21^.

The chemical structure of triacylglycerols (TAGs) has a significant effect on their stability and bioavailability in the gastrointestinal tract. The oxidation induction period of soybean oil has been shown to be delayed by 2–3 days through the addition of monoacylglycerol with DHA at the *sn*-2 position^19^. Camacho Paez et al.^18^ showed that locating essential fatty acids at the *sn*-2 position can improve their absorption during digestion.

No degradation was observed when the diacylstigmasterylcarbonoyl-*sn*-glycerols with the stigmasterol linked to the *sn*-2 position were kept at 60 °C, but degradation was 18% for compounds with the stigmasterol at the *sn*-3 position^23^. Barriuso et al.^24^ demonstrated that the presence of unsaturated fatty acids in lipid matrices has a protective effect on phytosterols during heating.

#### Degradation of fatty acids

The results of the simulated storage test on the oxidative stability of fatty acids in DASStGs is presented in Fig. 2B. When palmitic or oleic acids were linked at the *sn*-3 position of glycerol, their degradation ranged from 8 to 11%. When fatty acids were located at *sn*-2, only 1–3% of the palmitic and oleic acid content was lost. The position of fatty acids in the glycerol backbone was more significant than the unsaturation level or chain length. Similar results were obtained by Wang et al.^25^, who found that soybean oil, which contains a high amount of linoleic acid at the *sn*-2 position, was more resistant to autoxidation than structured soybean oil, which had a similar total fatty acid composition but a different positional distribution of linoleic acid. The position of fatty acids in the glycerol backbone also affects the nutritional properties of oils. The location of γ-linolenic acid at *sn*-2 position is important in determining the biological and clinical efficacy of borage oil^26^. Fatty acids at the *sn*-2 position appear to have an effect on cholesterol levels rather than on total fatty acids of triglycerides^27^.

#### Degradation of the stigmasterol part

Figure 2C shows the degradation of stigmasterol as a component of DASStGs while being kept at 60 °C for eight hours. The smallest decrease in stigmasterol level was seen for DO2SSt, where it amounted to 13%; the degradation of the other DASStGs ranged from 22 to 27%. These results show that the position of stigmasterol in the glycerol backbone has a significant effect on its degradation and that the *sn*-2 position is preferred. The degree of saturation of fatty acids had less of an effect on the loss of sterol than its position in the structure of the glycerol molecule.

When the temperature of free stigmasterol and stigmasteryl palmitate was maintained at 60 °C, about 3% and 6% of stigmasterol degraded^21^. When distigmasterol-modified acylglycerols were similarly kept at 60 °C, the decrease in sterol content ranged from 3 to 14%; when a saturated fatty acid formed part of these compounds, the sterol degradation was found to be half as large as in the compounds containing oleic acid^21^.

When diacylstigmasterylcarbonoyl-*sn*-glycerols were kept at 60 °C with stigmasterol in the *sn*-2 position, its degradation was not observed but acylglycerols containing stigmasterol at the *sn*-3 position degraded from 16 to 21%, depending on the fatty acids in molecule^23^.

#### Oxystigmasterols

Stigmasterol oxidation products (StOPs) were detected in all samples held at 60 °C; results are shown in Fig. 2D. The smallest total content of these compounds was 0.11 mg/g for DP2SSt and 0.14 mg/g for DO2SSt. When the stigmasterol was linked to the *sn*-3 position, the StOP content was higher at 0.36 mg/g for DP3SSt and 1.39 mg/g for DO3SSt.

Four oxidized derivatives of stigmasterol were identified in the heated samples: 7αOHSt, 7βOHSt, βEpSt, and 7ketoSt. The first two were detected in all samples, but 7αOHSt was found only in DASStGs with stigmasterol at the *sn*-3 position, while the βEpSt was noted only in DO3SSt. The most toxic oxidized derivative, triolSt was not identified in any of these samples.

Monitoring the phytosterol oxidation products (POPs) that form during food product storage is more important than monitoring the daily intake of phytosterol-enriched foods^10^. Animal studies have shown that absorption of POPs is much higher than that of non-oxidized sterols, and that 7hydroxy-derivatives were are relevant in plasma than 7keto-sterols, which are the typical major oxyphytosterols found in food products^14,28^.

When free stigmasterol and its esters with oleic and linoleic acids were kept at 60 °C for twelve hours, the total content of oxyphytosterols were 1.1, 0.2, and 9.7 mg/g, respectively^15^. The StOP content of distigmasteryl-modified acylglycerols maintained at this temperature for eight hours was found to be 1.4 mg/g for derivatives with stigmasterol attached by a carbonate linker and 0.4–0.5 mg/g for derivatives with stigmasterol attached by a succinyl linker^21^.

#### Fragmented molecules and oligomers

Keeping DASStGs at a temperature of 60 °C for eight hours did not lead to the formation of fragmented molecules or oligomers, the absence of which indicates that the samples would be stable during storage, which could be of great relevance when using these compounds as functional food additives.

Fragmented molecules and oligomers were noted when free sterols, their esters, and acylglycerols modified with stigmasterol were maintained at 60 °C^13,15,21^, but these were rather compounds with lower molecular weights than monomers; they included volatile compounds and stigmasteryl formate or hemisuccinate were detected^13,21^.

### Alterations of DASStGs when heated to 180 °C

#### Stability of DASStGs

**Figure 3:**
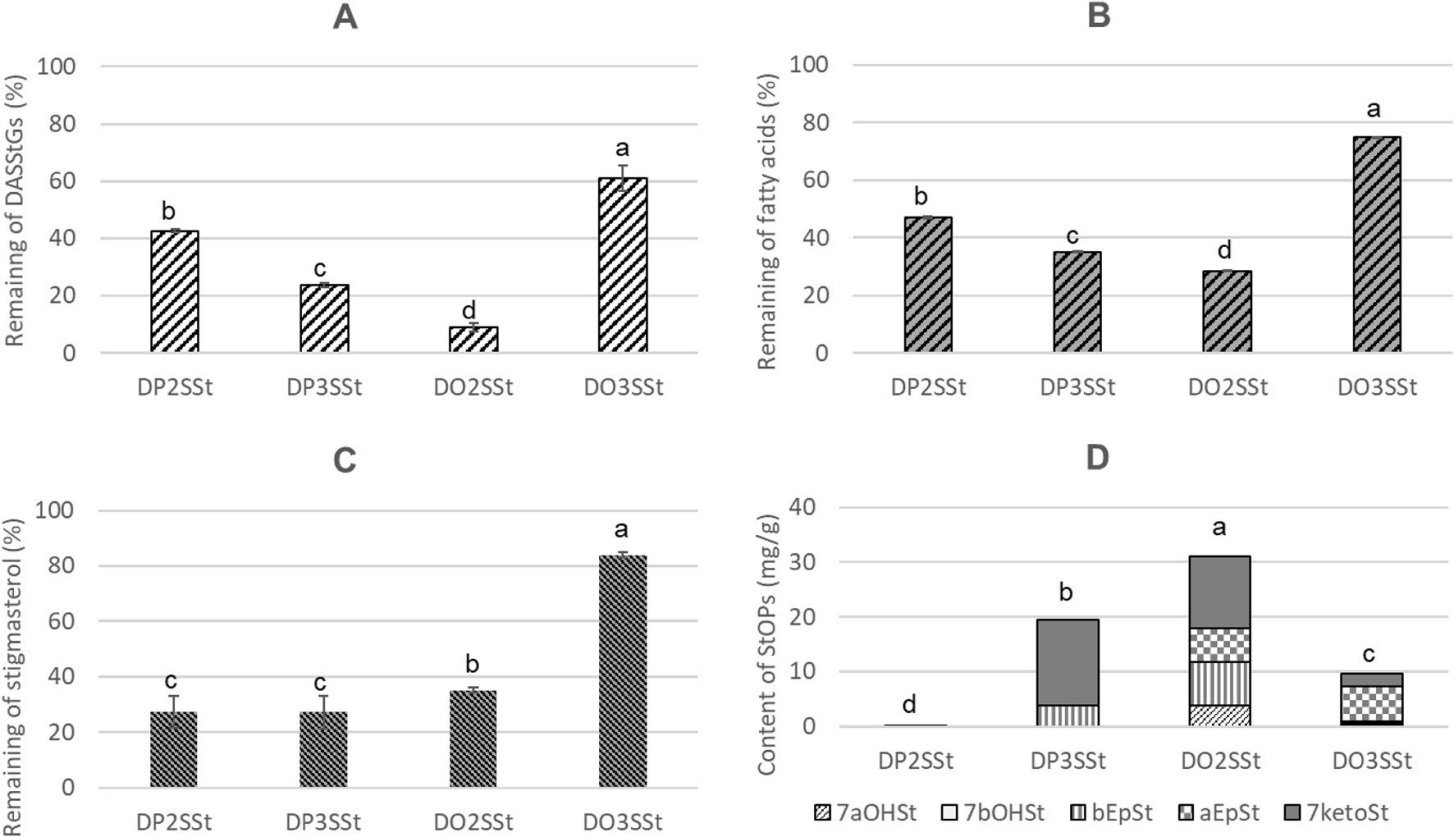
Remaining DASStGs (**A**), fatty acid parts (**B**), and stigmasterol parts (**C**), and the content of stigmasterol oxidation products formed after maintenance of DASStGs at 180 °C for eight hours; 7aOHSt: 7α-hydroxystigmasterol; 7bOHSt: 7β-hydroxystigmasterol; bEpSt- β-epoxystigmasterol; aEpSt: α-epoxystigmasterol; 7ketoSt: 7-ketostigmasterol; Means with different letters differ significantly (*p* < 0.05).

The level of degradation affecting DASStGs after being held at 180 °C for eight hours ranged from 39% for DO3SSt to 91% for DO2SSt (Fig. 3A). When two palmitic acid molecules were bonded with a glycerol backbone, the degradation was 57% for DP2SSt and 76% for DP3SSt. The differences in thermo-oxidative stability of the compounds were significant and may be due to the position of fatty acids and stigmasterol in the glycerol skeleton, as well as the saturation level of fatty acids. However, further research is needed to confirm this.

Almost 85% of 2,3-distigmasterylcarbonyl-1-oleoyl-*sn*-glycerol degraded when it was maintained at 180 °C for eight hours, but only 20% of 2,3-distigmasterylsuccinoyl-1-oleoyl-*sn*-glycerol content degraded under the same conditions^21^.

The relative thermo-oxidative stability of triacylglycerols depends on the unsaturation level of fatty acids and their position in the glycerol backbone, but not on the carbon chain length^17,25^. Our results showed that it also depends on the linker used to bond the stigmasterol to the glycerol skeleton, and compounds with a succinyl linker were more stable than those with a carbonate linker.

#### Degradation of fatty acids

The degree of degradation of the fatty acid part was correlated neither with the number of double bonds in the acid structure nor with their position in the triacylglycerol. The smallest decrease in the fatty acid part was noted for DO3SSt, where it amounted to 25% (Fig. 3B). When palmitic acid was linked at *sn*-1 and *sn*-2 positions of DASStGs, the degradation was much higher, at 65%. The opposite relationship was observed when the fatty acid residue was located at the *sn*-1 and *sn*-3 positions. When maintained at a temperature of 180 °C for eight hours, 53% of DP2SSt and 71% of DO2SSt was degraded. While being held at this temperature, the fatty acid residues underwent a number of reactions due to oxidation, polymerization, and decomposition.

Phytosterols chemically act as antioxidants^29^ and antipolymerizers^30^, and their presence in the structure of triacylglycerol may have various effects on the transformation of the whole molecule–a matter which requires further research.

There is lack of information on the effects of acyl group position in the glycerol backbone on oxidation and degradation during long term heating at frying temperature. Previous studies have generally compared the stability of oils and fats, which have different fatty acid compositions and positions in glycerol^31,32^. The structure of triacylglycerols may be among the most important factors determining their thermal stability. The mixture of saturated (PPP) and unsaturated (LLL) triacylglycerols was more susceptible to thermal oxidation at 150 °C and 180 °C than PPP/PLL and PPL^33^. The oxidation rates of LnLnL and LLLn were greater than LnLLn and LLnLn^34^. When 1,2-distigmasterylsuccinoyl-3-palmitoyl-*sn*-glycerol and 2,3- distigmasterylsuccinoyl-1-oleoyl-*sn*-glycerol were kept at 180 °C for eight hours, the degradation of fatty acids amounted to 40% and 6%, respectively^21^. The data shows that the chemical structure of triacylglycerols has crucially affects their stability. New structured acylglycerols may play an important role due to their technological and nutritional value.

#### Degradation of the stigmasterol part

The degradation of stigmasterol when heated at 180 °C was different than at 60 °C, and ranged from 16% for DO3SSt to 73% for both the acylglycerols with palmitic acid (DP2SSt and DP3SSt) (Fig. 3C). In the case o fDO2SSt, 65% of stigmasterol was degraded.

When free stigmasterol was held at 180 °C for eight hours, the degradation rate was about 90%, while for stigmasterol esters with palmitic and oleic acids the degradation of the stigmasterol moiety was 50–53%^21^. Holding stigmasteryl stearate, oleate, linoleate, and linolenate at the same temperature for twelve hours gave a degradation rate that ranged from 55 to 97%^22^. Higher unsaturation caused faster degradation, indicating that free radicals stimulate degradation of the sterol part of the molecule and affect the decomposition of the sterol^22^. Distigmasterol-modified acylglycerols with palmitic acid residue degraded slower than did stigmasteryl esters, with the degradation rate being 38% for 1,2-distigmasterylsuccinoyl-3-palmitoyl-*sn*-glycerol and 46% for 2,3- distigmasterylsuccinoyl-1-oleoyl-*sn*-glycerol^21^. When stigmasterol is incorporated into a glycerol molecule, its degradation is affected not only by the degree of saturation of the fatty acids, but also by its position in the glycerol backbone. The effect of fatty acid position in triglycerides on their thermal oxidative stability and dietary properties were determined^33^, but the incorporation of sterol in glycerol was examined only for asymmetric distigmasterol-modified acylglycerols^21^. The linker plays an important role in the stability of stigmasterol in distigmasterol-modified acylglycerols, and compounds with a succinyl linker showed greater stability than those with a carbonate linker.

#### Oxystigmasterols

The total quantity of stigmasterol oxidation products (StOPs) formed when DASStGs was held at 180 °C for eight hours ranged from 0.1 to 31.0 mg/g. The smallest amount of StOPs was formed in DP2SSt and the highest amount in DO2SSt (Fig. 3D). When 2,3-distigmasterylsuccinoyl-1-oleoyl-*sn*-glycerol and 1,2 distigmasterylsuccinoyl-3-palmitoyl-*sn*-glycerol were held at 180 °C for eight hours, the total quantity of StOPs was 0.8 and 4.1 mg/g, respectively^21^. Any analysis of total oxysterols will be incomplete if the composition of this fraction is not determined, with particular attention being paid to the triol derivative, which is considered the most toxic. The triolSt level was not determined in our samples. Only 7αOHSt was identified in DP2SSt, but βEpSt and 7ketoSt were detected in DP3SSt. The samples with oleic acid were found to contain four or five oxidation derivatives, with DO2SSt containing 7αOHSt, βEpSt, αEpSt, and 7ketoSt and DO3SSt additionally containing 7βOHSt.

The oxidation of sterols is a free-radical chain reaction that begins with the formation of hydroperoxides. Double bonds easily undergo radical attack, followed by hydrogen atom abstraction from the carbon atoms in the α-positions of the double bonds. Such allylic hydrogen atoms can easily be abstracted due to the relatively low C–H bond dissociation enthalpy. The final oxidation products of sterols are hydroxy, keto, and epoxy compounds^35^. We measured the cytotoxicity, proinflammatory, and proatherogenic effects of the phytosterol oxidation products^16^: When stigmasteryl esters with different fatty acids were held at 180 °C for twelve hours, oxyphytosterol level, which depends on the unsaturation level of fatty acids bonded with the sterol, initially increased and subsequently decreased^22^. The sterol oxidation products form through ring and side-chain free-radical reactions and can interact with each other. The small quantity of oxyphytosterols may be caused by the formation of dimers, oligomers, and low-weight molecular compounds^13^.

#### Fragmented molecules and oligomers

The formation of low-weight molecules, dimers, and oligomers of phytosterols after heating was determined first by Rudzińska et al.^13^, and have also been detected in the esters of plant sterols after thermo-oxidation^15^. These compounds affect the nutritional quality and biological properties of food products that contain them.

After heating the DASStGs, the samples were separated into the polar, mid-polar, and non-polar fractions and dimers, oligomers and fragmented molecules using HPLC.

DO2SSt contained 3% of polar dimers, 36% mid-polar dimers, and 29% mid-polar fragmented molecules; this was the most unstable compound. On the other hand DO3SSt was the most stable sample and was found to contain non-dimers and fragmented molecules. The composition of DP2SSt and DP3SSt was similar, containing 15% and 16% mid-polar dimers and 28% and 22% mid-polar fragmented molecules, respectively. No trimers or other oligomers were detected in the samples.

We determined the trimers and tetramers in free sitosterol that had been held at 120 °C and 180 °C for twenty-four hours^13^. After holding stigmasteryl oleate and stigmasteryl linoleate at a temperature of 180 °C for eight hours, the proportions of polar trimers, dimers, and non-polar dimers were detected as 13%, 21%, and 2% and 14%, 63%, and 2%, respectively^15^. When triacylglycerols with palmitic (P) and linoleic (L) acids were held at 150 °C for eight hours, the largest amount of polymers was determined for PPP/LLL (2:1), followed by PPL and PPP/PLL (1:1), and it was concluded that the thermal stability of triacylglycerols, such as edible oils, depends on their fatty acid composition^33^. Our results also showed that the position of the fatty acids affects the stability and that sterols can have a protective effect on unsaturated fatty acids.

### Cytotoxicity and genotoxicity

**Figure 4:**
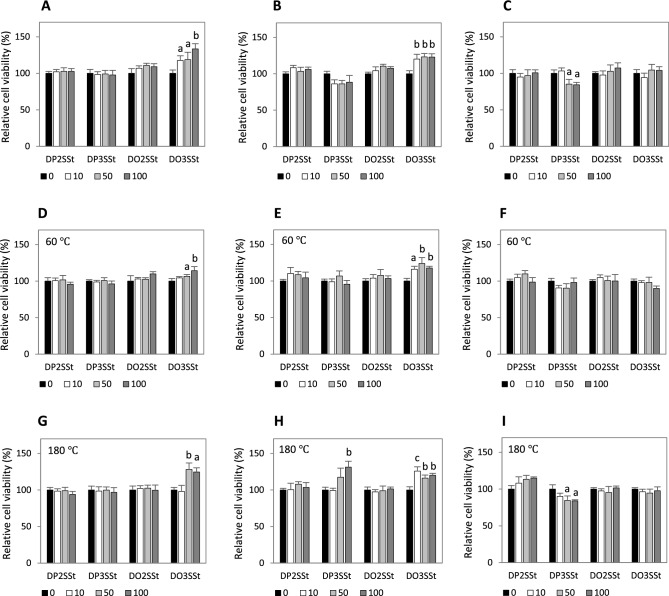
Cytotoxicity of 1,3-dipalmitoyl-2-stigmasterylsuccinoyl-*sn*-glycerol (DP2SSt), 1,2-dipalmitoyl-3-stigmasterylsuccinoyl-*sn*-glycerol (DP3SSt), 1,3-dioleoyl-2-stigmasterylsuccinoyl-*sn*-glycerol (DO2SSt), and 1,2-dioleoyl-3-stigmasterylsuccinoyl-*sn*-glycerol (DO3SSt), both unheated (**A**, **B**, **C**) and heated at 60 °C (**D**, **E**, **F**) and 180 °C (**G**, **H**, **I**), to human normal small intestinal epithelial FHS 74Int cells (**A**, **D**, **G**), colon mucosa CCD 481CoN cells (**B**, **E**, **H**) and liver THLE-2 cells (**C**, **F**, **I**). The cells were treated with these compounds at concentrations of 10 µg/mL, 50 µg/mL, and 100 µg/mL for 48 h. Cell viability determined by the MTT test is expressed relative to untreated control cells. ^a^*p* ≤ 0.05, ^b^*p* ≤ 0.01, ^c^*p* ≤ 0.001 significant differences in cell viability compared to control cells.

The effects of DP2SSt, DP3SSt, DO2SSt, and DO3SSt on the proliferation, viability, and metabolic activity of human cells derived from normal tissues of the digestive system are shown in Fig. 4. The experiments we performed failed to indicate any cytotoxic potential of these compounds on small intestine or colon mucosa cells (Fig. 4A and B). Also, thermo-oxidative treatment of the stigmasterol derivatives at 60 °C and 180 °C did not alter their cytotoxic potential on intestinal cells (Fig. 4D,E,G, and H). Moreover, DO3SSt at 10 μg/mL, 50 μg/mL, and 100 μg/mL stimulated the proliferation of small intestine cells (up to 34%) and colon mucosa cells (up to 23%) (Fig. 4A and B). The thermo-oxidation products of DO3SSt also enhanced the growth of intestinal FHS 74Int (up to 28%) and CCD 481CoN cells (up to 26%) (Fig. 4D,E,G, and H); however, the proliferation of FHS 74Int cells was significantly increased only when treated with heated DO3SSt at doses of 50 μg/mL and 100 μg/mL (Fig. 4D and G).

It is worth noting that DO3SSt held at 180 °C showed a higher capacity for FHS 74Int cell growth stimulation than did DO3SSt that had been held at 60 °C. DO3SSt that had been subjected to the thermo-oxidative process at 60 °C and 180 °C and introduced to the FHS 74 Int cell cultures at the maximum test concentration (100 μg/mL) led to increased cell proliferation by 14% and 25%, respectively (Fig. 4D and G). In the culture of colon CCD 481CoN cells treated with DO3SSt, cell proliferation increased independently of thermo-oxidative treatment (Fig. 4B, E, H).

The unheated stigmasterol derivatives did not affect THLE-2 liver cell proliferation or viability, except for DP3SSt, which decreased hepatocyte proliferation and viability when applied at 50 μg/mL (↓14%) and 100 μg/mL (↓15%) doses. DP3SSt at a lower dose of 10 μg/mL did not inhibit THLE-2 cell growth (Fig. 4C). Similar cytotoxic potential was observed for DP3SSt held at 180 °C under oxidative conditions (Fig. 4I). In contrast, when DP3SSt was held at 60 °C, its cytotoxic potential reduced. No cytotoxic effects were noted in the THLE-2 hepatocyte cultures treated with DP3SSt held at 60 °C (Fig. 4F).

**Figure 5:**
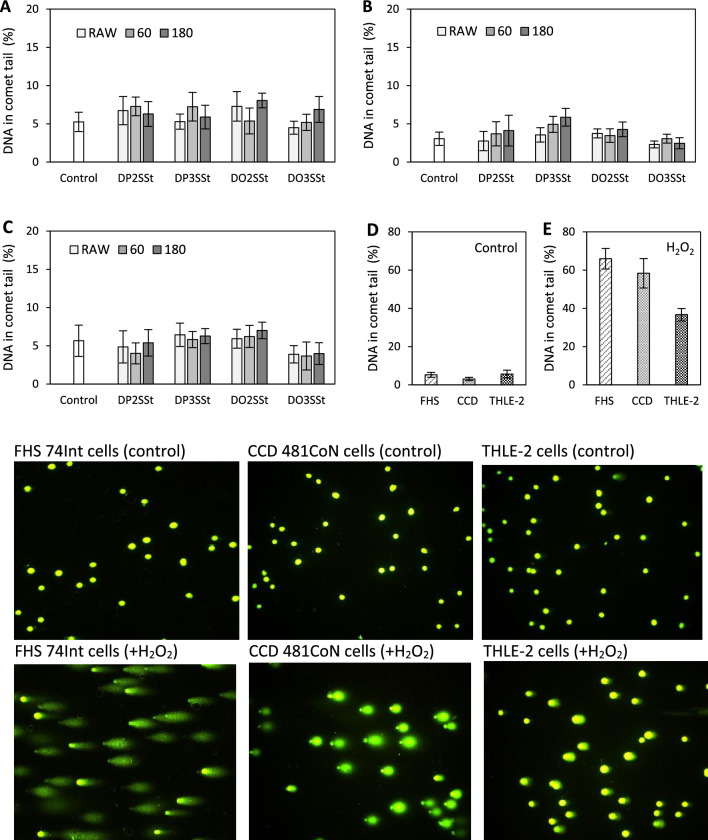
DNA strand breaks in normal small intestinal epithelial FHS 74Int cells (**A)**, colon mucosa CCD 481CoN cells (**B**), and liver THLE-2 cells (**C**) treated with 1,3-dipalmitoyl-2-stigmasterylsuccinoyl-*sn*-glycerol (DP2St), 1,2-dipalmitoyl-3-stigmasterylsuccinoyl-*sn*-glycerol (DP3SSt), 1,3-dioleoyl-2-stigmasterylsuccinoyl-*sn*-glycerol (DO2SSt), and 1,2-dioleoyl-3-stigmasterylsuccinoyl-*sn*-glycerol (DO3SSt), not held and held at 60 °C and 180 °C for eight hours. The cells were treated with the compounds at a concentration of 100 µg/mL for 48 h. DNA damage was expressed as the percentage of DNA content in the comet tail in the comet assay. The negative control consisted of the untreated cells (**D**) while the cells exposed to 100 µM H_2_O_2_ for 30 min to induce DNA damage were the positive control (**E**). ^a^*p* ≤ 0.05: significant differences in DNA damage compared to control cells.

The small intestinal FHS 74Int cells, colon CCD 481CoN cells, and liver THLE-2 cells were analyzed using an alkaline comet assay to detect DNA strand breaks induced in individual cells by treatment with the stigmasterol compounds. Figure 5 shows the DNA damage in the FHS 74Int (Fig. 5A), CCD 481CoN (Fig. 5B), and THLE-2 (Fig. 5C) cells treated for 48 h with the compounds at the maximum employed dose (100 μg/mL). The data in Fig. 5A–C) document that the frequency of DNA strand breaks in the untreated cells and in the cells treated with the tested did not differ significantly. Similarly, the stigmasterol compounds that were thermally processed (at 60 °C and 180 °C) did not increase DNA damage in cells. In comparison, the incidence of DNA strand breaks in the untreated cells and in the cells treated with an oxidant (100 μM H_2_O_2_) are shown in Fig. 5D and E, respectively.

Additionally, the microscopic documentation in Fig. 5 shows images of the small intestine cells, colon cells, and hepatocytes with noninduced and H_2_O_2_-induced DNA damage. Based on these results, we conclude that the stigmasterol compounds do not have any genotoxic effects on normal human cells of the intestinal tract and liver.

## Conclusions

New structured acylglycerols with stigmasterol bonded by a succinyl linker to the glycerol backbone were synthesized, and their stability was tested when their temperature was maintained at 60 °C and 180 °C to simulate storage and frying.

The holding temperature of 180 °C resulted in greater degradation of the compounds than 60 °C. Only in the case of DO3SSt was the loss of sterols greater at the lower temperature (26%) than at the higher temperature (16%), which is probably related to the fact that oxidation reactions took place at 60 °C, whereas at 180 °C the compounds underwent rapid thermal degradation. In all the test samples, the compounds containing oleic acid in their structure were the most stable. At 60 °C this was DO2SSt, while at 180 °C it was DO3SSt.

When held at 60 °C for eight hours, the degradation of DO2SSt amounted to 11%, while that of oleic acid and stigmasterol was 3% and 13%, respectively. After holding at 180 °C, the DO3SSt content decreased by 39%, oleic acid content by 25%, and stigmasterol content by 16%.

These results show that the type of fatty acid in the molecule is more important than its position in the glycerol structure.

None of the DASStGs, either before or after heating, exhibited cytotoxic potential to the small intestine and colon mucosa cells. Moreover, DO3SSt can stimulate the proliferation of the small intestine cells and colon mucosa cells. It can be also concluded that these compounds do not show genotoxic effects on normal human cells of the intestinal tract or liver.

The new synthesized stigmasterol derivatives could be a very safe and stable source of phytosterols in the humans diet. However, their use requires additional research into their absorption in the human body and bioavailability.

## Materials and methods

### Materials

*N*,*O*-Bis(trimethylsilyl)trifluoroacetamide (BSTFA) with 1% TMCS, *tert*-butyl methyl ether (MTBE), ethyl acetate, methanol, *n*-hexane, dichloromethane, diisopropyl ether, toluene, anhydrous pyridine, trifluoroacetic acid, sodium methanolate, silica gel (70–230 mesh, high purity), and standards of stigmasterol (95%), 5α-cholestane, and ethyl heptadecanoate were purchased from Sigma-Aldrich (Merck KGaA, Darmstadt, Germany). Glyceryl heptadecanoate was obtained from Larodan (Sweden).

### Methods

#### Synthesis of diacylstigmasterylsuccinoylglycerols (DASStGs)

1,3-Dipalmitoyl-2-stigmasterylsuccinoyl glycerol (DP2SSt) and 1,3-dioleoyl-2-stigmasterylsuccinoyl glycerol (DO2SSt) were synthesized from 2-benzyloxy-1,3-propanediol and dihydroxyacetone, respectively. 1,2-Dipalmitoyl-3-stigmasterylsuccinoyl-*sn*-glycerol (DP3SSt) and 1,2-dioleoyl-3-stigmasterylsuccinoyl-*sn*-glycerol (DO3SSt) were synthesized from 1,2-*O*-isopropylidene-*sn*-glycerol. Details of the synthetic protocols are described in Gładkowski et al.^36^ Fig. 1 shows the chemical structures of these compounds.

#### Heating of samples

The synthesized compounds were placed into glass vials filled with oxygen to ensure an oxygen atmosphere during thermal treatment. The samples were heated for eight hours at 60 °C, a temperature that simulates accelerated storage^37^, and at 180 °C to simulate the frying process. The experiment was performed in two replicates.

#### Degradation of heated DASStGs

The extent to which the DASStGs degraded depended on the stability of the fatty acids and stigmasterol. The decrease in DASStGS, stigmasterol, and fatty acids were therefore all determined separately.

The extent of DASStGs degradation was determined according to the method of Rudzińska et al.^21^ Briefly, once the samples had been dissolved in DCM, they were separated on an HPLC-ELSD Agilent 1260 Infinity II (Agilent, Santa Clara, CA, USA) equipped with an EC-C18 InfinityLab Poroshell 120 column operating in isotherm at 44 °C. The mobile phase consisted of acetonitrile (A) and DCM (B) with flow rate of 0.5 mL min^-1^, programmed as follows: 0 min 80% A and 20% B for 30 min, then changed to 55% A and 45% B; the phase composition was changed back to the initial parameters after 80 min and this was maintained for 10 min. The temperature of the evaporator and nebulizer was 30 °C, the gas flow rate was 1.6 L min^-1^ (SLM), and the photomultiplier tube (PMT) gain was 1.0. Glyceryl heptadecanoate was used as the internal standard. Each analysis was performed in three replications.

The AOCS Official Method Ch 6-91^38^ was used to determine the sterol part. Briefly, the samples were saponified and the unsaponifiables were silylated with BSTFA + 1% TMCS. The derivatives were separated on a gas chromatograph (an HP 6890 equipped with a DB-35MS capillary column programmed to 100 °C for 5 min, increased to 250 °C at 25 °C min^-1^, held for 1 min, then raised to 290 °C at 3 °C min^-1^ and held for 20 min). The FID temperature was set at 300 °C. Hydrogen was used as carrier gas with a flow rate of 1.5 mL min^-1^. 5α-cholestane was added as an internal standard to the samples prior to the analysis. Stigmasterol was identified by comparison with the retention time of the standard. Each samples was analyzed in triplicate.

The degree of degradation of fatty acids was determined following the AOCS Official Method Ce 1 k-07^39^,. Briefly, the samples were hydrolyzed and then methylated with boron trifluoride in methanol. To separate the fatty acid methyl esters, a Trace 1300 gas chromatograph equipped with an SP-2560 capillary column and an FID was used. Hydrogen was the carrier gas, supplied at a rate of 1.5 mL min^-1^. The oven temperature was programmed to increase from 160 °C to 220 °C at a rate of 12 °C min^-1^, and was held there for 20 min. The injector and detector temperatures were both set to 240 °C. Fatty acid methyl esters were identified by comparison with the standard retention times. Methyl heptadecanoate was used as the internal standard. Each analysis was performed in duplicate.

#### Stigmasterol oxidation products (StOPs)

The concentration of stigmasterol oxidation products (StOPs) was determined according to the procedure described by Raczyk et al.^22^ Briefly, samples of DASStGs before and after heating were treated with sodium methoxide. After 2 h at room temperature, the organic fraction was extracted with chloroform and fractionated with SEP-PAK NH_2_ using hexane and hexane:MTBE. Finally, the StOPs were removed with 7 mL of acetone, derivatized using BSTFA + 1% TMCS, and analyzed on a Hewlett-Packard 6890 gas chromatograph equipped with a DB-5MS column (50 m × 0.2 mm × 0.33 μm; J&W, Folsom, CA). The column temperature was programmed to an initial 160 °C held for 1 min, and then increased at 40 °C min^-1^ to 270 ºC and held for 1 min; it then rose at 4 °C min^-1^ to 280 °C, with the final temperature being held for 25 min. The injector worked in splitless mode. Hydrogen was the carrier gas and was supplied at a flow rate of 1 mL min^-1^.

The StOPs were identified using an Agilent Technologies 7890A GC system coupled to a quadrupole 7000 QQQ-MSD. The column and conditions described above were used again. All mass spectra were recorded in electron impact ionization mode using an energy of 70 eV, and the masses were scanned from 100 to 700 Da. The ion source was held at 200 °C, while the injector was held at 300 °C. The NIST Mass Spectra Library, in combination with our own laboratory library of collected sterol data and retention data on standards, was used to identify the compounds. Samples from an autonomous series were analyzed in triplicate.

#### Oligomers

The samples were divided into polar, mid-polar, and nonpolar fractions using silica gel (Sigma-Aldrich, silica gel 60, 63–200 µm)^40^. Briefly, after heating, the DASStGs were dissolved in toluene and applied to a silica gel column. The nonpolar fraction was eluted with a mixture of hexane and diisopropyl ether (82:18, v/v), the mid-polar fraction was eluted with diisopropyl ether, and then polar fraction was eluted with chloroform:methanol (2:1, v/v). The purity of all fractions and the accuracy of separation was verified thin-layer chromatography. A silica gel TLC plate was developed with hexane:diisopropyl ether (82:18, v/v), sprayed with a copper sulphate–phosphoric acid–methanol solution and held at 120 °C.

The oligomer composition was determined by HPLC on two Phenogel columns (100 Å i500 Å, 5 µL, 300 × 7.8 mm; Phenomenex, Torrance, CA, USA) connected in series. The column temperature was at 30 °C, the light scattering detector was at 30 °C, the detector pressure was 2.5 bars, and the injection volume was 1 µL. The liquid phase was dichloromethane (DCM) at a flow rate of 1 mL min^-1^.

#### Cytotoxicity and genotoxicity experiments

The cytotoxicity and genotoxicity of the stigmasterol derivatives (DP2SSt, DP3SSt, DO2SSt, and DO3SSt), as well as that of their thermo-oxidative degradation products and of the oxidized derivatives that formed under heat treatment at 60 ℃ and 180 ℃ in an oxygen atmosphere, were determined in normal human cells from the digestive system, including the small intestine FHs 74 Int (ATCC CCL241), colon mucosa CCD 841CoN (ATCC CRL-179), and liver epithelial THLE-2 (ATCC CRL-2706) cells obtained from the American Type Culture Collection (ATCC, Manassas, VA, USA). Cell lines were cultured according to ATCC recommendations. In the cytotoxicity experiments, the cells were seeded in 96-well plates at a density of 1.5 × 10^4^ cells/cm^2^ (CCD 841CoN and FHs 74 Int cell lines) and 2.0 × 10^4^ cells/cm^2^ (THLE-2 cell line). The 24-h cell cultures were treated for 48 h with unheated and heated stigmasterol derivatives at concentrations of 10 μg/mL, 50 μg/mL, and 100 μg/mL. The MTT assay was used to determine cell viability and metabolic activity in the cell cultures exposed to the compounds. The detailed procedure for the MTT test has been described in previous cytotoxicity studies^41^.

The genotoxicity of the stigmasterol compounds was analyzed using the single-cell gel electrophoresis (SCGE) comet assay. This method detected the DNA strand breaks in normal human cells induced by treatment with unheated and heated stigmasterol derivatives. The cells were grown in six-well plates at established density and standard culture conditions and were then exposed to the test compounds for 48 h. The untreated cells and the cells that had been treated with H_2_O_2_ (100 μM, 30 min) to induce oxidative DNA damage were taken as negative and positive controls, respectively. The comet assay followed a previously published protocol^42^. Briefly, the harvested cells were suspended in low melting-point agarose and placed on microscope slides precoated with agarose of normal melting-point. Cell lysis at 4 °C, alkaline electrophoresis (pH > 13), and neutralization (pH 10) were performed sequentially. The slides were stained with SYBRGold (molecular probes) and viewed under a fluorescence microscope (Axiovert 200, Zeiss, Carl Zeiss, Gottingen, Germany). The cells were analyzed for DNA damage using CometScore software (TriTek Corp., Sumerduck, VA, USA). Data on DNA strand breaks were expressed as mean percentages of DNA content in the comet tail. At least a hundred cells were analyzed on a microscope slide; Three hundred cells were considered for DNA damage detection in one sample.

#### Statistical analysis

The experiments and analysis were performed in three independent replicates and the data presented here are mean values with standard deviations (± SD). The statistical analysis was performed using Statistica version 13.3 (Statsoft, Tulsa, OK, USA). The significance of the main effects was determined by one-way analysis of variance (ANOVA). The equality of variances assumption was verified using Levene’s test. The parametric Tukey’s post hoc test was used to determine differences between the mean values of multiple groups. The statistical significance was considered at *p* < 0.05. RStudio (version 2022.07.01 + 554 with packages FactoMineR v.2.4 and factoextra v.1.0.7) was used for principal components analysis (PCA).

## Data Availability

The datasets used and analysed in this are available from the corresponding author upon reasonable request.

## Abbreviations

DASStG: Diacylmonostigmasterylsuccinoyl-*sn*-glycerol
DP2SSt: 1,3-Dipalmitoyl-2-stigmasterylsuccinoyl-*sn*-glycerol
DO2SSt: 1,3-Dioleoyl-2-stigmasterylsuccinoyl-*sn*-glycerol
DP3SSt: 1,2-Dipalmitoyl-3-stigmasterylsuccinoyl-*sn*-glycerol
DO3SSt: 1,2-Dioleoyl-3-stigmasterylsuccinoyl-*sn*-glycerol
BSTFA: *N*,*O*-Bis(trimethylsilyl)trifluoroacetamide
TMCS: Trimethylchlorosilane
MTBE: *tert*-Butyl methyl ether
TFA: Trifluoroacetic acid
DCM: Dichloromethane
StOPs: Stigmasterol oxidation products
TriolSt: Stigmastentriol
7ketoSt: 7-Ketostigmasterol
7αOHSt: 7α-Hydroxystigmasterol
7βOHSt: 7β-Hydroxystigmasterol
αEpSt: α-Epoxystigmasterol
βEpSt: β-Epoxystigmasterol
PCA: Principal component analysis
PSs: Phytosterols
POPs: Phytosterol oxidation products
TAGs: Triacylglycerols
P: Palmitic acid
O: Oleic acid
L: Linoleic acid
Ln: Linolenic acid
